# Fachkräfteentwicklung in der Rheumatologie: Ein berufsstruktureller Überblick und gesundheitspolitischer Weckruf

**DOI:** 10.1007/s00393-021-01012-4

**Published:** 2021-05-18

**Authors:** Ellen Kuhlmann, Luzia Bruns, Kirsten Hoeper, Torsten Witte, Diana Ernst, Alexandra Jablonka

**Affiliations:** 1 Klinik für Rheumatologie und Immunologie, Medizinische Hochschule Hannover, Carl-Neuberg-Str. 1, 30625 Hannover, Deutschland; 2 Regionales Kooperatives Rheumazentrum Niedersachsen e. V., Hannover, Deutschland

**Keywords:** Fachkräfteentwicklung, Rheumatologie, Berufsstatistiken, Altersgruppenspezifische Trends, WHO National Health Workforce Accounts, Geschlechterspezifische Trends, Deutschland, Health workforce development, Rheumatology, Profession statistics, Age-group related trends, WHO National Health Workforce Accounts, Gender-specific trends, Germany

## Abstract

**Hintergrund und Fragestellung:**

Fachkräftemangel in der Rheumatologie in Deutschland ist als Versorgungsproblem erkannt. Die Gesundheitspolitik hat mit neuen Planungszielen reagiert, aber es fehlen effektive Interventionsstrategien. Ziel dieser Studie ist ein systematischer berufsstruktureller Überblick, um die Grundlage für Interventionen zu verbessern und Möglichkeiten für ein effektives Fachkräftemanagement aufzuzeigen.

**Methode:**

Die WHO National Health Workforce Accounts (NHWA) dienen als konzeptioneller Rahmen. Ausgewählt werden 4 Indikatoren: Personalbestand, Arbeitsmarktbewegungen, Komposition und Weiterbildung. Die Exploration von Entwicklungstrends stützt sich auf vergleichende Analysen von Altersgruppen und Zeitreihen. Die Erhebung nutzt öffentliche Statistiken und andere Sekundärliteratur; die Auswertung erfolgt deskriptiv.

**Ergebnisse:**

In Deutschland sind 1076 Ärzt*innen mit einer Facharztqualifikation oder Schwerpunktbezeichnung in der internistischen Rheumatologie ärztlich tätig. Die absolute Zahl verdoppelte sich seit 2000 deutlich (91 %), aber mit einem demografischen Bias. Im Zeitraum 2000 bis 2019 stieg die Zahl der über 50-Jährigen deutlich, aber die der unter 50-Jährigen nur um 9 %; seit 2010 sind die Zahlen in der Gruppe 40 bis 50 Jahre rückläufig. Im Jahr 2019 waren mehr Rheumatolog*innen im Rentenalter als unter 40-Jährige ärztlich tätig. Seit 2015 schwächt sich der steigende Trend insgesamt ab, aber am stärksten im Krankenhaussektor; die Weiterbildungen lassen keine konstante Steigerung erkennen.

**Schlussfolgerungen:**

Berufsstrukturelle Trends zeigen, dass die gesundheitspolitischen Planziele mit den verfügbaren Humanressourcen nicht zu erreichen sind. Gefordert ist ein besseres Fachkräftemanagement, insbesondere durch Innovation der Weiterbildung, Aufgabenverschiebung und verbesserte Geschlechtergerechtigkeit.

## Abstract (en)

**Background and objective:**

Health workforce shortage in German rheumatology has been identified as a healthcare service and delivery problem. Health policy has increased staffing targets, yet effective intervention strategies are lacking. This research aimed to systematically map the rheumatology workforce to improve the evidence for interventions and explore possibilities for more effective health workforce management.

**Methods:**

The WHO National Health Workforce Accounts provided a conceptual framework for the mapping exercise. Four major sets of indicators were selected, comprising staffing levels, health labor market flows, composition and education/training. A comparison of age groups and time series was applied to explore trends. Public statistics and other secondary sources served our analysis using descriptive methodology.

**Results:**

In Germany there are 1076 physicians specialized in internal medical rheumatology. Absolute numbers have nearly doubled (91%) since 2000 but with a strong demographic bias. Between 2000 and 2019 numbers markedly increased in the group aged 50 years and older but only by 9% in the younger group under 50 years; since 2010 the group aged 40–50 years even faces a decrease. In 2019, the absolute numbers of rheumatologists in retirement age exceeded those aged 40 years and under. Since 2015 an expanding workforce trend has overall flattened but this was strongest in the hospital sector; the numbers in resident training did not show any relevant growth.

**Conclusion:**

Health workforce trends reveal that an available number of rheumatologists cannot meet new health policy planning targets. There is a need for effective health workforce management, focusing on innovation in resident training, improved task delegation and gender equality.

Zunehmende Unterversorgung und Nachwuchsprobleme in der internistischen Rheumatologie sind bekannt, und die Fachgesellschaften und Standesvertretungen mahnen schon länger nachdrücklich zum Handeln [5, 11–14, 22, 25, 28, 31, 38, 39]. Mit der Festsetzung neuer Planziele für die Zahl der Rheumatolog*innen durch den Gemeinsamen Bundesausschuss (G-BA) sind zwar erste gesundheitspolitische Reaktionen zu erkennen [16], es bleibt aber die Frage: „Woher nehmen?“ Was wissen wir über die Fachkräftesituation in der Rheumatologie in Deutschland? Hier setzt unsere Untersuchung an und versucht, einen systematischen Überblick zu geben.

Bisher spielen hoch spezialisierte kleine Fachgebiete wie die Rheumatologie auch international kaum eine Rolle in den Debatten um Fachkräftesicherung und verbessertes Management der Personalressourcen; das gilt für die Politik wie für die Forschung [23, 30]. Diese Fachgebiete sind aber von einem steigenden Fachkräftemangel im Gesundheitssystem in besonderer Weise betroffen. Lange Ausbildungszeiten und eine insgesamt niedrige Zahl von Leistungserbringern und Weiterbildungsstätten lassen kaum Spielraum für zeitnahe Lösungen des Fachkräftemangels. Hinzu kommen eine unübersichtliche Datenlage und unzureichende Monitoringsysteme.

Als gesundheitspolitische Reaktion auf das zunehmende Missverhältnis zwischen steigendem Bedarf und verfügbaren Personalressourcen erfolgte eine Erhöhung der Planungsziele. Der G‑BA hat 2019 für die Rheumatologie eine Mindestquote von 8 % der internistischen Facharztgruppen mit einer Erhöhung auf 10 % bis 2025 als Ziel definiert [16]. Er hat aber nicht gesagt, wie dieses Ziel erreicht werden könnte. Angemessene gesundheitspolitische Interventionsstrategien zur Lösung der berufsstrukturellen Probleme sind bisher nicht erkennbar. Vielmehr konzentriert sich die Debatte primär auf den Versorgungs*bedarf *(oder ökonomisch gedacht, die „Nachfrage“), ohne das Pendant, das verfügbare *Angebot* an Qualifikationen, systematisch zu berücksichtigen. Für die Rheumatologie in Deutschland heißt das: Ein systematisches Fachkräftemanagement findet entweder gar nicht oder auf Basis einer unzureichenden Datenlage statt.

Diese Situation beinhaltet potenzielle Risiken und Nachteile für die Versorgung der Bevölkerung [38]. So können Patent*innen möglicherweise nicht oder nicht zeitnah von den diagnostischen und therapeutischen Innovationen profitieren [29, 38]. Die Zunahme chronischer Erkrankungen und der demografische Wandel [12, 37] werden den Versorgungsbedarf weiter erhöhen, und die COVID-19-Pandemie hat die Probleme nochmals verschärft [8, 18]. So drängt die vermutlich noch länger anhaltende Krisensituation die mühsam errungene Aufmerksamkeit für chronische Erkrankungen [37] erneut in den Hintergrund. Zudem erzeugt die Pandemie neue gesundheitliche Risikolagen für Menschen mit rheumatischen Erkrankungen und auch für deren Behandelnde. Hierdurch wird der seit Längerem existierende Versorgungsengpass nochmals verstärkt, und die beruflichen Belastungsfaktoren der Rheumatolog*innen werden erhöht.

Während die Versorgungsfragen vergleichsweise gut dokumentiert sind, sind systematische Informationen zur Fachkräftesituation in der Rheumatologie eher rar. Differenziertere Informationen liegen v. a. aus Nordamerika vor, die auch Fragen von Qualifikationsmix, Aufgabenverschiebungen und Subspezialisierungen sowie systematischere Ansätze des Fachkräftemanagements untersuchen [2, 3, 6, 26, 32]. Auf europäischer Ebene hat die Fachgesellschaft (EULAR) das Thema in den Fokus gerückt und richtungsweisende Positionspapiere zur Fachkräfteplanung in der Ärzteschaft vorgestellt [9, 34]. Darüber hinaus wurde ein breiteres Spektrum neuer Herausforderungen thematisiert. Insbesondere liegen Studien vor zur Aufgabenverschiebung und zu der Rolle der Pflegekräfte [4] (2020), zu Bedarfslagen der jüngeren Generation [10, 15] und zu geschlechterspezifischen Beschäftigungsmustern und Diskriminierungen [1, 27].

In Deutschland wird v. a. der Mangel an Ärztinnen und Ärzten hervorgehoben [16, 38]. Seit Kurzem wird jedoch das Potenzial von Skill-mix-Ansätzen in Bezug auf die Aufgabendelegation in einem Modellprojekt „Rheumatologische Fachassistenz“ systematischer erforscht [19, 21, 39]. Ein weiteres, bereits seit Längerem intensiver beleuchtetes Thema sind die Defizite in der Weiterbildung [5, 11, 22, 25, 28, 31]. Braun et al. haben 2011 darauf hingewiesen, dass nur etwa 65 % der Weiterbildungsermächtigten tatsächlich ausbilden [5]. Aktuelle Daten liegen nicht vor, obschon es sich hier um einen sehr zentralen Indikator zur Abschätzung der berufsstrukturellen Entwicklung handelt, der für die Bewertung und realistische Umsetzung neuer Planungsziele herangezogen werden muss.

Untersucht wurden auch die Karriereziele und die Arbeitssituation der Rheumatolog*innen; die Informationen basieren jedoch auf einem kleinen, regional begrenzten Sample [28]. Geschlechterspezifische Fragen werden hingegen in der deutschen Debatte (bestenfalls) marginal adressiert. Sprachlich kommen Frauen nach wie vor meist gar nicht vor, und eine Sensibilität für Diskriminierung ist nicht erkennbar. Es mangelt zudem an weiblichen Rollenvorbildern auf der Leitungsebene; erst 2015 wurde die erste Frau auf einen Lehrstuhl für Rheumatologie berufen. Auch berufspolitisch sind Frauen als Entscheidungsträgerinnen insgesamt unterrepräsentiert, obschon es Hinweise auf Veränderungen gibt; systematische Informationen liegen hierzu bisher nicht vor.

Ziel der vorliegenden Studie ist es, einen differenzierten deskriptiven Überblick über die berufsstrukturelle Situation zu erstellen, um die Grundlage für Entscheidungen zu verbessern und zu einem effektiveren Fachkräftemanagement beizutragen. Es soll gezeigt werden, dass die berufsstrukturelle Perspektive eine zentrale Größe in der versorgungspolitischen Debatte werden muss, um die Grenzen der gegenwärtigen Bedarfsplanung und der Zielvorgaben sowie die Fehlentwicklungen sichtbar zu machen. Der Schwerpunkt liegt auf den internistischen Rheumatologinnen und Rheumatologen. Im Zentrum stehen 3 Fragestellungen.

- Wie stellt sich die Verteilung der Rheumatolog*innen dar, und welche Hinweise bieten sich auf Mangel- und Fehlverteilungen?
- Welche Kapazitäten stehen in der Weiterbildung zur Verfügung?
- Welche gesundheitspolitischen Handlungsempfehlungen lassen sich aus dem berufsstrukturellen Überblick ableiten?

## Methode

Das Untersuchungsdesign stützt sich auf die National Health Workforce Accounts (NHWA) [35, 36]. Die NHWA wurden vom Regionalbüro Europa der Weltgesundheitsorganisation (WHO) auf Basis umfassender Konsultationen mit Expertinnen und Experten der Mitgliedsstaaten entwickelt, um die Länder bei der Entwicklung von Monitoringsystemen und Strategien zur Verbesserung der Fachkräftesituation im Gesundheitssystem zu unterstützen. Sie bieten eine komplexe Toolbox von Indikatoren, die entsprechend der nationalen und professionsspezifischen Bedingungen flexibel angewendet werden können [36]. So steht ein systematischer Ansatz für die Analyse der Fachkräfteentwicklung zur Verfügung, der existierende Daten effektiv nutzen und unterschiedliche Informationsquellen kombinieren kann [24].

Die Datensammlung stützt sich überwiegend auf öffentliche Statistiken, ergänzt um Website Recherchen und andere Sekundärliteratur sowie Experteninformationen. Insbesondere das interaktive Portal der Gesundheitsberichterstattung [17] bietet Möglichkeiten für differenzierte Analysen. Untersuchungsfeld ist die Rheumatologie mit Fokus auf die Ärzt*innen für internistische Rheumatologie. Hierfür werden die Kategorien „Fachärzte“ (Innere Medizin und Rheumatologie und Innere Medizin SP Rheumatologie) und „Schwerpunkte“ zusammengefasst und als relevante Grundgesamtheit betrachtet; berücksichtigt werden nur die ärztlich Tätigen. Diese Grundgesamtheit wird nachfolgend als „Rheumatolog*innen“ bezeichnet (sofern nicht anders angegeben). Die verfügbaren Statistiken weisen für die Rheumatologie nur Kopfzahlen aus, aber bieten keine Informationen über Teilzeitbeschäftigung und Vollzeitäquivalente.

Inhaltlich liegt der Schwerpunkt auf Arbeitsmarkt- und Beschäftigungsmerkmalen und auf der Weiterbildung. Ausgewählt werden quantitative Indikatoren aus den 4 Gruppen „ärztlich tätige Rheumatolog*innen“, „Arbeitsmarktbewegung“, „Skill-mix-Komposition“ sowie „Weiterbildung“ (Tab. 1). Die Auswahl der Indikatoren beinhaltet die in der Kategorie 9 „workforce supply“ der „EULAR points to consider for the conduction of workforce requirement studies“ [9] genannten quantitativen Indikatoren (Tab. 1), aber geht darüber hinaus. Die Materialien wurden deskriptiv ausgewertet. Die Beschränkung auf quantitative Indikatoren erfolgt aufgrund der Datenlage für die Rheumatologie in Deutschland.

**Table 1:** 

Kategorien	Indikatoren
Bestand an aktivem Personal, ärztlich tätig	Dichte national, subnational/Bundesland, Ost/West
	Verteilung Altersgruppen
	Verteilung Geschlecht
	Migration (im Ausland geboren und im Ausland ausgebildet)
Arbeitsmarktbewegung	Arbeitsmarkteintritt
	Arbeitsmarktaustritt
	Ungleichgewicht (freie Stellen, Arbeitslosigkeit)
Skill-Mix	Sektorale Komposition
	Medizinische Fachgebiete
	Medizinische Fachangestellte
	Andere Gesundheitsberufe (Pflege/Fachpflege, Hausärzt*innen)
Weiterbildung	Akkreditierte Institutionen (bzw. Personen)
	Weiterbildungseintritt und -austritt

Quellen: eigene Auswahl, adaptiert von NHWA [35, 36]

Um Entwicklungstrends und Dynamiken zu erfassen, werden primär 3 Zeitpunkte ausgewählt: 2000, 2010 und 2019 (letzte verfügbare Daten). Wenn sinnvoll, werden ergänzend 2005 und/oder 2015 betrachtet, um die Effekte der Novelle der Weiterbildungsordnung 2003 sowie eine potenzielle Trendwende differenzierter zu erkennen. Die Indikatoren Fachärzt*innen Dichte, Altersgruppen und Geschlecht sowie die sektorale und disziplinäre Komposition werden vergleichend betrachtet; für ausgewählte Aspekte wird die Gesamtzahl der Ärzteschaft und die der Fachärzt*innen für Innere Medizin (ärztlich tätig) ergänzend als Vergleichsbasis herangezogen.

## Ergebnisse

### Bestand und Verteilung an Fachpersonal und Entwicklungstrends

Im Bundesgebiet waren 2019 insgesamt 1076 ärztlich tätige Rheumatolog*innen bei den Ärztekammern registriert, was einer Facharztdichte von 1,294 pro 100.000 Bevölkerung entspricht. Hier soll noch mal ausdrücklich darauf hingewiesen werden, dass es sich um berufsstrukturelle Zahlen handelt. Mit Blick auf die Versorgung ist die *Facharzt*dichte eine hypothetische Größe, die die maximale Kapazität angibt. Das ist hilfreich, um die Umsetzung politischer Bedarfsplanungen realistischer zu bewerten und die Lücken zwischen Bedarf und Angebot genauer zu beziffern. Für die *Versorgungs*dichte wurden deutlich niedrigere Zahlen ermittelt [12, 13, 38], die nicht Gegenstand unserer Untersuchung sind.

**Figure 1:**
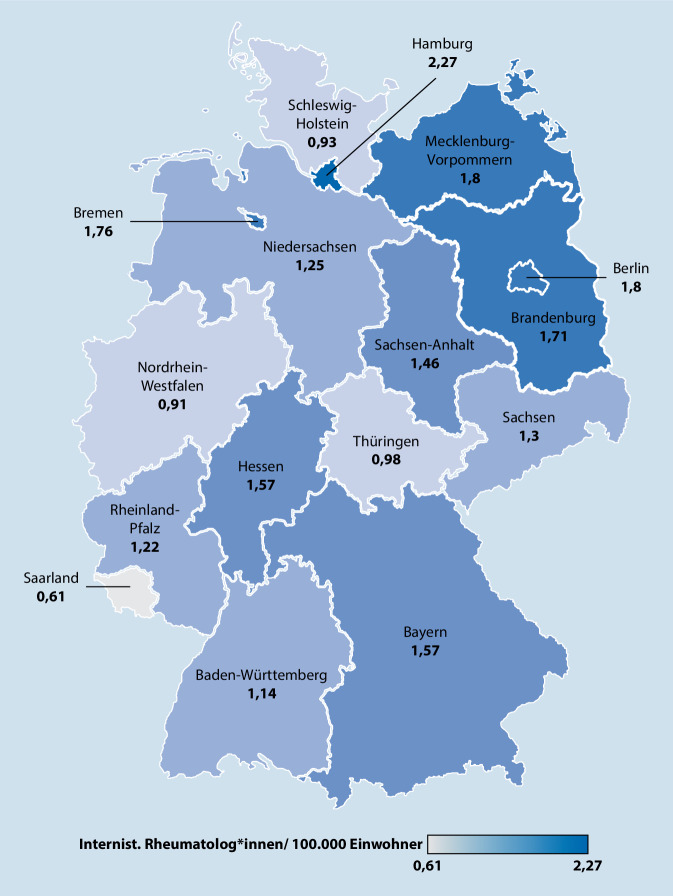

Unsere Analyse (Abb. 1) legt offen, dass die Lücke zwischen Bedarf und Angebot selbst bei Ausschöpfung sämtlicher verfügbarer Qualifikationen auf absehbare Zeit gar nicht zu schließen wäre. Die Fachgesellschaft gibt als Zielgröße für die Versorgungsdichte mindestens 1:50.000 an [38], aber umgerechnet auf diesen Maßstab stehen maximal nur 0,647 Fachkräfte zur Verfügung (Rheumatologe Facharztdichte). Zudem werden ausgeprägte regionale Ungleichverteilungen sichtbar, die sich keinem der bekannten Ost-West- und Nord-Süd-Muster zuordnen lassen.

Die absolute Zahl der Rheumatolog*innen hat sich von 2000 bis 2019 fast verdoppelt (+91 %) (Tab. 2), aber davon profitierten primär die höheren Altersgruppen ab 50 Jahre. Die Gruppe der unter 50-Jährigen weist insgesamt nur einen schwachen Zuwachs (+9 %) auf und die Gruppe der 40- bis 50-Jährigen ab 2010 sogar einen deutlichen Negativtrend; in absoluten Zahlen ging die Personalstärke im Zeitraum 2010 bis 2019 um 74 Rheumatolog*innen zurück. Im Jahr 2019 waren mehr Rheumatolog*innen im Rentenalter als unter 40-Jährige in der Versorgung tätig. Der beobachtete Trend insgesamt steigender Zahlen schwächt sich ab 2015 ab und stagniert nahezu im Krankenhaussektor. Dieser Trend ist möglicherweise noch stärker, da von einer Zunahme der Teilzeitbeschäftigung auszugehen ist. Gesicherte Daten liegen aber nicht vor.

**Table 2:** 

Altersgruppen	Mit ärztlicher Tätigkeit					Sektorale Verteilung, ambulant und stationär^a^																			
						Ambulant					Niedergelassen					Angestellt					Stationär				
–	2000	2005	2010	2015	2019	2000	2005	2010	2015	2019	2000	2005	2010	2015	2019	2000	2005	2010	2015	2019	2000	2005	2010	2015	2019
Alle	569	662	790	979	1076	222	291	389	480	561	220	284	343	385	413	2	7	45	95	148	315	327	351	441	454
< 35 Jahre	3	2	1	13	15	–	–	1	–	4	–	–	–	–	–	–	–	–	–	4	3	2	–	13	11
35 <40 Jahre	86	51	50	75	88	23	13	13	16	26	22	11	9	9	10	1	2	4	7	16	62	34	37	59	60
40 <50 Jahre	248	321	338	290	264	89	136	149	126	123	88	131	124	93	74	1	5	25	33	49	142	169	172	149	138
50 <60 Jahre	146	191	287	396	424	63	95	154	215	240	63	95	148	183	188	–	–	6	32	52	77	79	109	160	165
60 <66 Jahre	68	71	73	134	164	33	31	42	76	95	33	31	40	67	87	–	–	2	9	8	31	36	28	48	57
≥ 66 Jahre	18	26	41	71	121	14	16	30	47	73	14	16	23	33	54	–	–	7	14	19	–	7	5	12	23

Quelle: eigene Darstellung, GBE [17], abgerufen 20.02.2021

^a^Die Sektoren „Behörden etc.“ und „andere“ sind in der Darstellung nicht erfasst

Ein weiterer Unsicherheitsfaktor sind die Rheumatolog*innen, die weitere Facharztbezeichnungen führen. Im Jahr 2014 wurden z. B. 27 ambulant tätige Rheumatolog*innen mit Zulassung zur Versorgung in 2 Facharztgebieten und 123 Rheumatolog*innen mit gleichzeitiger hausärztlicher/allgemeininternistischer Zulassung ermittelt, bei 407 fachärztlich rheumatologisch niedergelassenen Ärzt*innen, was einem Anteil von 30 % nicht rein rheumatologisch tätigen Rheumatolog*innen entspricht [13].

**Figure 2:**
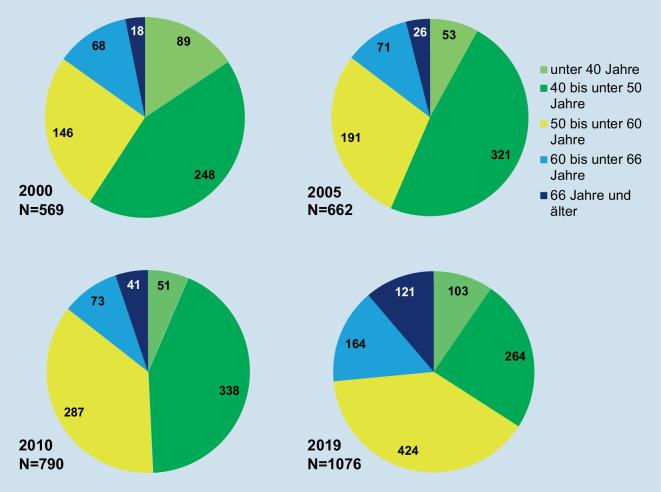

Ein Vergleich der Altersgruppen im Zeitverlauf deutet auf eine Trendwende in der demografischen Zusammensetzung der Rheumatolog*innen nach 2010 hin (Abb. 2; Tab. 2). Die bis 2010 zahlenmäßig stärkste Gruppe der 40- bis 50-Jährigen verzeichnete nach vorherigen Zuwächsen ab 2010 deutliche Verluste, die in den jüngeren Gruppen nicht vollständig kompensiert wurden. Der Anteil der unter 50-Jährigen war 2019 auf etwa ein Drittel der Fachgruppe gesunken; 2000 lag er noch bei 59 %, 2005 bei 57 % und 2010 bei 49 %. Die Daten deuten darauf hin, dass die Novelle der Weiterbildungsordnung die Spezialisierung offensichtlich nicht deutlich beschleunigen konnte; nur sehr wenige (2019, *n* = 15) erwerben vor dem 35. Lebensjahr die fachärztliche Anerkennung. Auch für die Gruppe der unter 40-Jährigen lagen die Zahlen 2019 noch unter dem Anteil der Rheumatolog*innen im Rentenalter.

**Figure 3:**
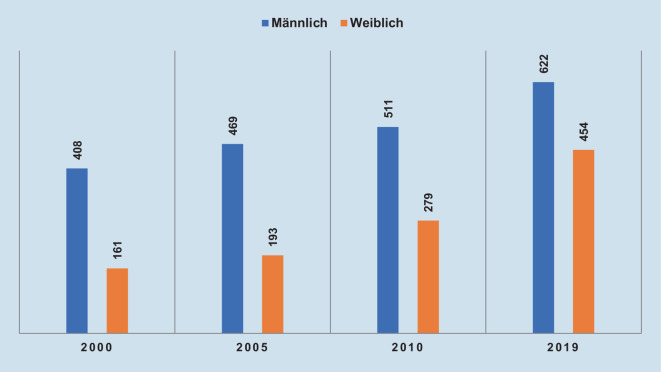

Im Geschlechtervergleich (Abb. 3) zeigen sich kontinuierlich steigende Frauenquoten von 28 % im Jahr 2000 auf 42 % Frauen im Jahr 2019, was einen allgemeinen Trend in der Ärzteschaft spiegelt (48 % Frauen, ärztlich tätig, 2019) [7]. Zwischen 2010 und 2019 ist die absolute Zahl der weiblichen Mitglieder deutlich stärker gestiegen als die der Männer, obschon beide Gruppen zahlenmäßige Zuwächse verbuchen konnten. Dieser Trend weist im Geschlechtervergleich auf eine höhere Präferenz von Frauen für die Rheumatologie hin. Im Vergleich mit der Inneren Medizin (39 % Frauen) [7] wird ein leicht überdurchschnittlicher Frauenanteil in der Rheumatologie (42 %) sichtbar.

Die verfügbaren Statistiken bieten keine Informationen über den Anteil ausländischer Fachkräfte in der Rheumatologie; das gilt für die Kategorien „im Ausland geboren“ und „Ausbildung im Ausland“. Zu vermuten ist aber, dass der Anteil eher unter dem Durchschnitt der Gruppe aller Fachärzt*innen liegt, da die primären Senderländer Rumänien und Syrien [7] sowie andere osteuropäische Länder allenfalls in sehr geringer Zahl über rheumatologische Fachkräfte verfügen. Ob und in welchem Umfang Mobilität in der Weiterbildung eine Rolle spielt, lässt sich nach den vorliegenden Daten nicht abschätzen. Als Rekrutierungsgruppen wären v. a. die Ärzt*innen aus Griechenland und Österreich zu betrachten, da diese Länder weit überdurchschnittliche Ärztezahlen und Engpässe in der Weiterbildung aufweisen.

### Arbeitsmarktbewegung

Die Zahl der jährlichen Neuzugänge in der Rheumatologie lag in den letzten Jahren zwischen 30 und 50 [38]; 2018 betrug sie 50, darunter 31 Frauen, und stieg 2019 auf 61 Neuzugänge (39 Frauen) an [7]. Die Zahl der jährlichen Abgänge aus der ärztlichen Tätigkeit ist nicht dokumentiert, es liegen aber keine Hinweise auf auffällige Bewegungen vor. Nach der BÄK-Statistik lassen sich 2019 insgesamt nur 24 rheumatologische Fachärzt*innen ohne ärztliche Tätigkeit berechnen (ohne diejenigen im Ruhestand und berufsunfähig); 2018 waren es 27 (eigene Berechnungen [7]). Demnach ist Arbeitslosigkeit eine zu vernachlässigende Größe, zumal sich in der Kategorie der nicht ärztlich Tätigen auch die temporären Ausfälle z. B. infolge von Elternzeit verbergen.

Ebenso deutet nichts auf zahlenmäßig relevante Abwanderungen ins Ausland hin. Diese sind ohnehin in der gesamten Ärzteschaft nur gering und nehmen seit 2014 kontinuierlich ab [7]. Mobilitätsbewegungen sind eher innerhalb der Medizin insbesondere zwischen den verschiedenen Subspezialisierungen der Inneren Medizin zu vermuten, doch lassen die Statistiken hierzu keine Aussagen zu. Ein Monitoring des Stellenmarktes existiert ebenfalls nicht, Expert*inneninformationen weisen aber auf ein Unterangebot an Bewerber*innen hin.

### Skill-Mix, sektoral und berufsgruppenspezifisch

**Figure 4:**
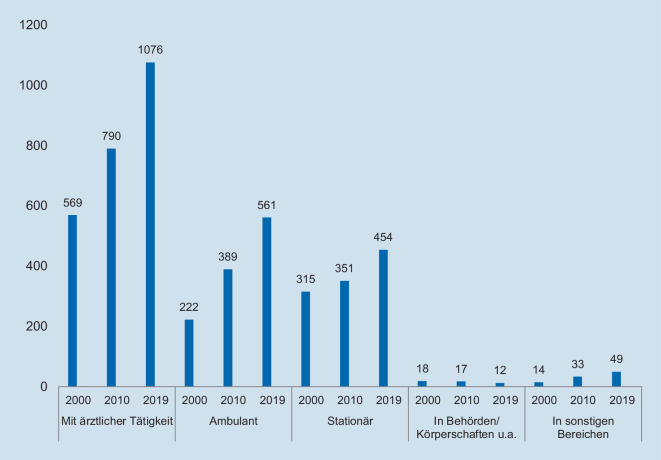

Die Mehrheit der Rheumatolog*innen war 2019 in der ambulanten Versorgung tätig (52 %) (Abb. 4), darunter etwa 74 % als Niedergelassene und 26 % im Angestelltenverhältnis. Auf die stationäre Versorgung entfielen 42 % und weitere 1,1 % auf „Behörden und Körperschaften“ sowie 4,6 % auf die nicht näher differenzierten „sonstigen Bereiche“ (Abb. 2). Zum Vergleich: In der Inneren Medizin lag der Anteil der Pro-Kopf-Zahl im stationären Sektor mit 45 % nur wenig höher [7].

Ein Vergleich der sektoralen Verteilung (Abb. 4) macht deutlich, dass der Zuwachs primär im ambulanten Sektor erfolgte, der von 2000 bis 2019 um etwa 153 % zulegte und eine kontinuierliche Steigerung aufweist. Im Vergleich dazu ist der positive Trend im Krankenhaussektor deutlich schwächer (+44 %). Der zahlenmäßig unbedeutende Sektor der „Behörden und Körperschaften“ nahm leicht ab, wohingegen die Kategorie „sonstige“ deutliche Zunahmen aufweist.

Mit Blick auf die professionsspezifische Komposition stellen die internistischen Rheumatolog*innen den größten und versorgungspolitisch relevanten Teil (Tab. 2). Ergänzend soll auf die Zusatzweiterbildung „Kinder-Rheumatologie“ hingewiesen werden, über die 193 Ärzt*innen verfügen (Daten 2019) [17]. Zuverlässige Informationen über weitere an der Versorgung beteiligte Gruppen liegen nur für die chirurgischen (orthopädischen) Rheumatolog*innen vor. Hier zeigte sich im Zeitraum 2000 bis 2019 ein kontinuierlicher und sehr deutlicher Rückgang der Gesamtzahl (508 vs. 364), der in der demografischen Betrachtung noch deutlicher wird: Die Gruppe der unter 50-Jährigen war 2019 auf 22 orthopädische Rheumatolog*innen gesunken [17]. Seit 2010 steht jedoch eine Zusatzweiterbildung „Orthopädische Rheumatologie“ zur Verfügung, die bisher von 130 Ärzt*innen erworben wurde (ärztlich tätig, Stand 2019; 2010 *n* = 47) [17].

Pflegekräfte spielen nur im Krankenhaussektor eine Rolle und können nicht fachspezifisch zur Rheumatologie zugeordnet werden. Anders als z. B. in den USA und Großbritannien ist bisher keine Spezialisierung als rheumatologische Fachpflegekraft möglich. Für den ambulanten Sektor und die Zielgruppe der medizinischen Fachangestellten steht bereits seit 2006 ein 60-stündiges Curriculum mit Zertifizierung DGRh/BDRh zum Abschluss „Rheumatologische Fachassistenz“ zur Verfügung. Ein neues 120-stündiges BÄK-zertifiziertes Curriculum „Fortbildungscurriculum für Medizinische Fachangestellte und Arzthelfer/innen Rheumatologie“ liegt der BÄK zur Zertifizierung vor. Die Entwicklungen werden gegenwärtig in einem überregionalen Forschungsprojekt koordiniert und beobachtet. Erste Studienergebnisse weisen auf ein neues Potenzial für die rheumatologische Versorgung hin [19, 21]. Allerdings sind die berufsstrukturellen Effekte noch nicht einzuschätzen; zudem werden Delegationsmodelle insgesamt in Deutschland bisher von nur etwa der Hälfte chronisch Kranker akzeptiert [20].

### Weiterbildung

**Figure 5:**
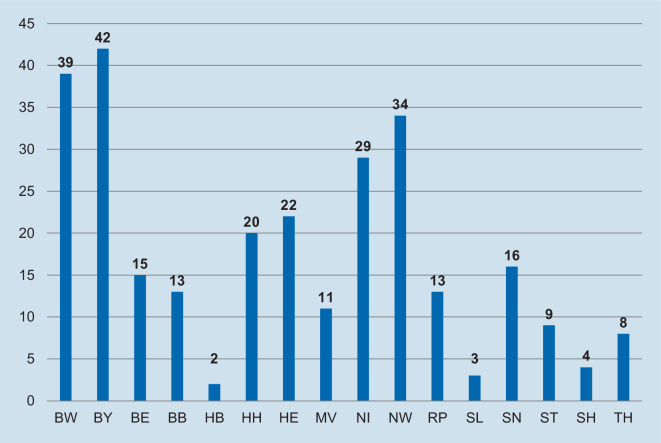

Ein Monitoring der Weiterbildung ist nicht etabliert. Information über die Zahl der Weiterbildungsermächtigten in der internistischen Rheumatologie können nur über eine Handsuche der Websites der verschiedenen Landesärztekammern zusammengetragen werden. Die Abb. 5 zeigt die absolute Zahl pro Bundesland. Insgesamt konnten so für Deutschland 280 Weiterbildungsermächtigte ermittelt werden (Stand November 2020).

Die vorliegenden Daten weisen aber mehrere Unsicherheitsfaktoren auf. Die tatsächliche Zahl könnte weitaus niedriger sein, da möglicherweise die Weiterbildungsermächtigten im Ruhestand ohne ärztliche Tätigkeit weiterhin auf den Websites erscheinen. Zu berücksichtigen ist auch, dass Universitätskliniken und große Praxen mehrere Weiterbildungsermächtigte beschäftigen. Die Zahl der Einrichtungen ist folglich deutlich niedriger als die Zahl der Weiterbildungsermächtigten. Für Niedersachsen ergab eine Handsuche z. B. ein Verhältnis von 29 Weiterbildungsermächtigten in 16 Einrichtungen (eigene Recherche). Weiter ist zu berücksichtigen, dass vermutlich ein relevanter Teil gar nicht ausbilden kann oder will, wie eine vor einigen Jahren von der Fachgesellschaft durchgeführte Untersuchung ergab [5]. Eine zuverlässige Einschätzung der tatsächlich vorhandenen Kapazitäten in der Weiterbildung ist auf Basis der gegenwärtig verfügbaren Daten nicht möglich.

Für die Weiterbildungsassistent*innen ist die Datenlage noch weitaus schlechter. Selbst die Handsuche über Websites der Landesärztekammern und der Einrichtungen führt hier nicht weiter. Wie für Niedersachsen exemplarisch geprüft wurde, machen nur wenige Einrichtungen Angaben zur Anzahl der Weiterbildungsassistent*innen. Weder die Akkreditierung noch der Verbleib unterliegen einem systematischen Monitoring. Auf dieser Grundlage wird jede Planung zum Blick in die Glaskugel.

## Diskussion und Perspektiven

Wir haben argumentiert, dass die Fachkräftesituation in der Rheumatologie berufsstrukturell und nicht nur versorgungspolitisch betrachtet werden muss, um auf Fehlentwicklungen besser reagieren zu können. Mit den NHWA haben wir einen systematischen methodischen Rahmen vorgestellt. Die Ergebnisse liefern differenziertere Informationen zum Missverhältnis zwischen Bedarf und Angebot, zu regionalen Ungleichheiten und zu sektoralen, altersgruppen- und geschlechterspezifischen Verteilungen. Sie bieten Hinweise auf prospektive Entwicklungen und machen wesentliche Schwachstellen und Lücken in den vorliegenden Daten sichtbar.

Die Ergebnisse weisen aufgrund veränderter Trends in den jüngeren Berufsgruppen und steigender Bedarfslage auf Dynamiken hin, die den existierenden Fachkräftemangel und die Versorgungsdefizite zukünftig erheblich verstärken könnten. Bereits jetzt liegt die rheumatologische Facharztdichte sehr deutlich unter dem vom G‑BA und der Fachgesellschaft und Berufsvertretung ermittelten Bedarf [16, 38]. Wenn selbst die hypothetische Größe der maximal verfügbaren Qualifikationen weit unter der Bedarfsplanung liegt, so gilt dies vor dem Hintergrund von Teilzeitstellen, Doppelqualifikationen und Tätigkeit außerhalb der Patientenversorgung umso mehr für die real für die Versorgung verfügbare Kapazität. Diese Lücken in der Versorgung sind gesundheitspolitisch v. a. deshalb relevant, da große Teile der Bevölkerung betroffen sind und von einer Verbesserung im Zugang und der Zugänglichkeit zur Versorgung profitieren würden [12, 38].

Was sind die Treiber für die sinkenden Zahlen? Die Veränderungen gehen wesentlich von den jüngeren Altersgruppen aus, die deutlich niedrigere Zuwachsraten im Vergleich zu den älteren Gruppen aufweisen. Der altersgruppenspezifische Trend weist auf strukturelle Defizite in der Weiterbildung hin, denn trotz exzellenter medizinischer Entwicklungen und guter ökonomischer Bedingungen nimmt offensichtlich die Attraktivität der Rheumatologie ab. Dieser Trend muss geschlechterspezifisch betrachtet werden, da die Zuwächse in der Gruppe der Frauen höher sind als in der Gruppe der Männer. Bisher ist nicht untersucht, ob sich hieraus veränderte Anforderungen an die Arbeitsorganisation, die Arbeitszeitgestaltung und die Karriereplanung ergeben und wie hierauf angemessen reagiert werden könnte. Möglicherweise ergeben sich veränderte Arbeitszeitpräferenzen in der jüngeren Altersgruppe und/oder in der Gruppe der Frauen im Vergleich zu den Männern, die bei der Fachkräfteplanung sowie im Fachkräftemanagement berücksichtigt werden müssen.

Ebenso wenig sind die Gründe und die Auswirkungen der sinkenden Attraktivität des stationären Sektors angemessen untersucht. Dieser Trend ist nicht nur im Hinblick auf die Versorgung relevant. Vielmehr zeichnen sich hier aufgrund der Aus- und Weiterbildungsaufgaben, insbesondere der Universitätskliniken, unmittelbare Auswirkungen auf die Personalkapazitäten ab, die zukünftig zu einer weiteren Verschlechterung der Fachkräftesituation führen wird. Die Weiterbildung der internistischen Rheumatolog*innen wird aktuell auch dadurch gefährdet, dass weniger als die Hälfte aller Universitäten einen rheumatologischen Lehrstuhl haben [31] und nur 8 von 37 medizinischen Fakultäten einen Lehrstuhl für Rheumatologie unterhalten.

Die Ergebnisse unserer Untersuchung machen erhebliche gesundheitspolitische Defizite sichtbar. Die Zielvorgabe einer deutlichen Erhöhung der Mindestquote rheumatologischer Fachärzt*innen in der ambulanten Versorgung [16] ist ohne grundlegende und systematische Interventionen nicht zu erreichen [31]. Zu Erinnerung: Auf Basis der registrierten Rheumatolog*innen ergibt sich ein Verhältnis von 0,647:50.000 Bevölkerung, der Versorgungsbedarf der Bevölkerung wird aber auf 1:50.000 geschätzt. Neue gesundheitspolitische Planungsvorgaben allein können das Problem fehlender Fachkräfte demzufolge nicht lösen, da das Angebot gar nicht vorhanden ist. Im stationären Sektor sind die gesundheitspolitischen Defizite noch offensichtlicher, da bisher nichts auf eine angemessene Wahrnehmung der Problemlagen hindeutet.

Welche Möglichkeiten lassen sich identifizieren für Interventionen? Ein erster notwendiger Schritt wäre es, ein effektives Fachkräftemanagement der Rheumatologie als gesundheitspolitisch relevante Aufgabe zu erkennen, Verantwortung zu übernehmen und entsprechende Ressourcen bereitzustellen. Dazu gehören transparente Daten sowie eine insgesamt verbesserte Daten- und Forschungslage. Die Einführung eines systematischen Monitorings der Weiterbildungskapazitäten und -verläufe wäre ein Schritt in diese Richtung. Das gilt für einen Überblick über die Zahlen als Grundbedingung jeder Fachkräfteplanung, aber mindestens ebenso für die qualitativen Indikatoren, wie z. B. die Arbeitsbedingungen, Karrierechancen, Abbau von Geschlechterdiskriminierungen und mehr weibliche Rollenvorbilder in Leitungspositionen sowie bessere Informationen zu den insgesamt veränderten Bedarfslagen und Bedürfnissen in jüngeren Altersgruppen. Auch die Gründe für die abnehmende Attraktivität der Rheumatologie in der Gruppe der jüngeren Männer erfordern stärkere Beachtung und gezielte Interventionsmaßnahmen.

Insgesamt sind kurzfristige Maßnahmen aufgrund der spezifischen Situation der Rheumatologie als kleines Fach mit langen Ausbildungszeiten nur sehr begrenzt zielführend. Nachhaltige Lösungen erfordern längerfristige Perspektiven. Ansatzpunkte für Veränderungen bieten sich v. a. durch Innovationen in der Weiterbildung und im stationären Sektor, das Angebot von ausreichenden stationären Weiterbildungsstellen sowie durch eine verbesserte Rekrutierung jüngerer Altersgruppen und den Abbau geschlechterspezifischer Benachteiligungen.

Innovationen in diesen Bereichen sind dringend notwendig und langfristig zielführend. Sie allein werden aber das Dilemma der fehlenden rheumatologischen Fachkräfte kurz- und mittelfristig nicht lösen können, wie das eklatante Missverhältnis zwischen Bedarfszahlen und Personalressourcen (Angebot) zeigt. Gefordert sind komplexere Strategien, die berufsgruppenspezifische Innovationen in der Rheumatologie mit einer verbesserten Nutzung der Kompetenzen anderer Berufsgruppen (s. z. B. Modellprojekte zu rheumatologischer Fachassistenz) und Veränderungen in der Weiterbildung und Arbeitssituation verbinden.

### Limitationen

Die Studie legt den Schwerpunkt auf quantitative berufsstrukturelle Indikatoren und nutzt Sekundärliteratur für die Analyse, insbesondere öffentlich zugängliche Berufsstatistiken und Informationen offizieller Websites. Hierdurch ist es möglich, Trends aufzuzeigen sowie Lücken in der vorhandenen Berichterstattung aufzudecken. Die hoch aggregierten Daten können jedoch keine Auskunft geben über individuelle und qualitative Indikatoren, wie z. B. die Arbeitsorganisation und die beruflichen Präferenzen. Die Ursachen von Mangel und Fehlverteilungen der Fachkräfte können mit dem methodischen Ansatz und den verfügbaren Daten nur ansatzweise aufgedeckt, aber nicht systematisch erfasst werden. Hierzu bedarf es weiterer Untersuchungen und der Erhebung von Primärdaten. Das gilt in ähnlicher Weise für versorgungsbezogene Fragen. Wie beschrieben, erfolgte diese Untersuchung aus der Fachkräfteperspektive. Sie liefert eine verbesserte Grundlage für versorgungsbezogene Bedarfsplanungen, die aber eigene Studien erfordern.

Weitere Limitationen ergeben sich durch die fehlenden Daten zu Vollzeitäquivalenten bzw. Teilzeitquoten sowie durch die unklaren Zuordnungen bei Doppelqualifikationen und Tätigkeit in unterschiedlichen Sektoren. Da zukünftig von einer Zunahme hybrider Beschäftigungsformen sowie neuer transsektoraler Versorgungsmodelle auszugehen ist, die sich mit den bisherigen statistischen Kategorien nicht abbilden lassen, zeichnen sich hier grundlegend neue Herausforderungen für das Monitoring der Gesundheitsfachkräfte ab.

## Fazit für die Praxis

- Ein Vergleich zwischen dem Angebot an Fachkräften, die bei den Ärztekammern registrierten Rheumatolog*innen, und dem prognostizierten Bedarf (G-BA) macht einen hohen Mangel sichtbar; die Schere wird sich zukünftig weiter öffnen.
- Bei insgesamt steigenden Zahlen weisen die höheren Altersgruppen wesentlich stärkere Zuwächse auf als die jüngeren. Rheumatolog*innen im Rentenalter stellen eine zahlenmäßig größere Gruppe in der Versorgung als die unter 40-Jährigen.
- Im ambulanten Sektor hat sich die Zahl der Rheumatolog*innen erhöht, im Krankenhaussektor ist der Zuwachs deutlich niedriger; hierdurch werden auch die Weiterbildungskapazitäten im Krankenhaussektor reduziert.
- Das gesundheitspolitische Ziel einer höheren Mindestquote in der ambulanten rheumatologischen Versorgung ist ohne grundlegende Innovationen in der Weiterbildung und im stationären Sektor nicht zu erreichen.
- Gefordert sind gesundheitspolitische Verantwortung und die Entwicklung eines systematischen Fachkräftemanagements für die Rheumatologie, das Innovationen in der Weiterbildung mit Aufgabenverschiebung und verbesserter Geschlechtergerechtigkeit verbindet.
